# Pleural effusion following ventriculopleural shunt: Case reports and review of the literature

**DOI:** 10.4103/1817-1737.65048

**Published:** 2010

**Authors:** Elif Küpeli, Cem Yilmaz, Şule Akçay

**Affiliations:** *Department of Chest Diseases, Baskent University School of Medicine, Ankara, Turkey*; 1 *Department of Neurosurgery, Baskent University School of Medicine, Ankara, Turkey*

**Keywords:** Hydrocephalus, pleural effusion, ventriculo-pleural shunt

## Abstract

Ventriculo-pleural shunt (VPLS) is an acceptable alternative in the management of hydrocephalus. Imbalance between the production and absorption of cerebrospinal fluid an lead to formation of pleural effusion in patient with VPLS and on occasion produce symptoms. Pleural effusion could be a transudate or a non-specific exudate. We report our experience with this modality in relation to formation of pleural effusion and review the literature to make recommendation for its management. Information related to patients’ demographics, smoking history, prior pulmonary and occupational history, indication, duration and complications of the VPLS and their management was gathered to substantiate current recommendation with our experience.

A variety of modalities exists for the management of hydrocephalus.extracranial shunts are routinely used to divert cerebrospinal fluid (CSF) into the extravascular compartment for the palliation of symptoms from hydrocephalus. Ventriculo-peritoneal (VPS) and ventriculo-atrial (VAS) shunts are the most widely used methods for this indication. Ventriculo-pleural shunt (VPLS) has also been used as an alternative to the peritoneal and atrial shunts since 1954.[1] It is considered for draining CSF in selected patients when conventional sites are not suitable either due to adhesions, infection, thrombosis or obliteration. Studies have suggested that VPLS is an acceptable alternative for draining CSF in children as well as among adults.[2–7]

The most common complication following VPLS placement is pleural effusion.[8,9] We retrospectively reviewed charts of all five patients who underwent VPLS placement at our institution. The purpose of our study was to study the outcomes and pulmonary complications associated with the VPLS. Information related to patients’ demographics, smoking history, prior pulmonary and occupational history, indication, duration and complications of the VPLS and their management was gathered. Following collection of the data, review of the literature was carried out by studying all the articles listed on the PubMed with keyword VPLS in their title published in English language during the last 50 years.

## Case Reports

### Case 1

A 67-year-old female, a life-long non-smoker was admitted with right ptosis and was diagnosed with macroadenoma of the pituitary gland. She suffered with essential hypertension but had no prior pulmonary history. Seven years following the initial surgery, i.e. at the age of 74, a recurrence of the adenoma was detected and she underwent repeat resection. On day 10, after the surgery, she developed hydrocephalus and a VPLS was placed. The pleural location was chosen in view of the prior history of peritonitis. Five days following the shunt placement, she was found to be short of breath. On physical examination, dullness over the right hemithorax and crackles over the base were evident. Partial pressure of oxygen was 42 mmHg on the arterial blood gases, on room air. Chest X-ray (CXR) revealed a large right pleural effusion. At thoracentesis, 1500 ml of transudative fluid was drained [Table 1]. Microbiological studies on the fluid remained negative. She required two more thoracentesis, each at 3 weeks interval for the recurrence of large pleural effusion, and on each occasion 1500 cc of transudative fluid was removed. After the third thoracentesis, she remains symptom free without recurrence of the pleural fluid. The VPLS devise remains in its original position fully functional and without any complications for the follow-up of two years period.

### Case 2

**Figure 1:**
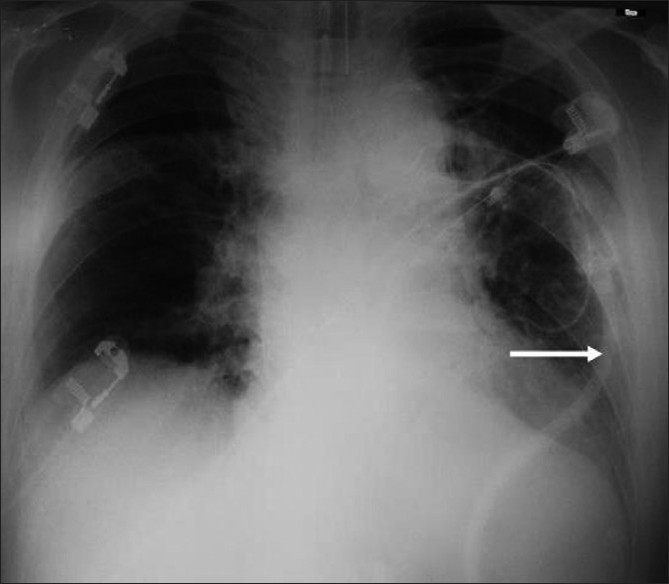
Chest X-ray of case 2 after the VPLS placement. Arrow shows the ventriculo-pleural shunt

**Figure 2:**
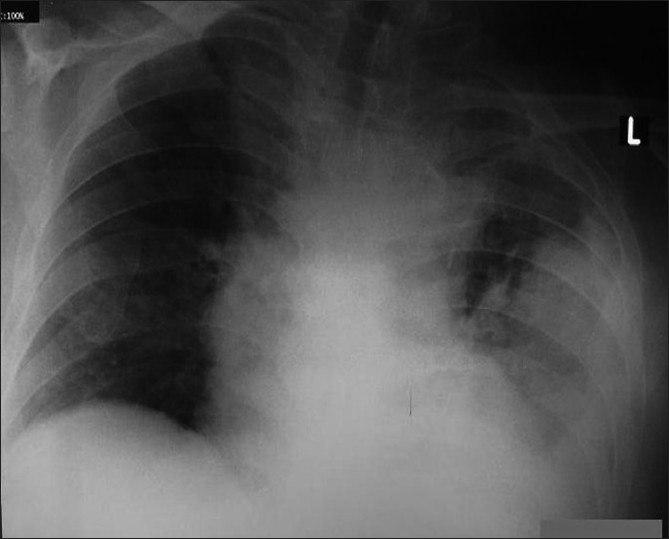
Chest X-ray of the case 2. left-sided large pleural effusion after the VPLS.

A 75-year-old female underwent lumber stabilization surgery for the weakness of the lower extremities. During the surgery, a dural leak was observed. Initially a VPS was inserted; however, it had to be removed due to infection and hence a VPLS was considered [Figure 1]. Twenty days following the shunt placement, she developed shortness of breath and was diagnosed with massive left-sided pleural effusion [Figure 2]. She was afebrile. The fluid was an exudate with a lymphocytic predominance. Microbiological studies on the fluid remained negative. The pleural fluid adenosine deaminase level was within normal limits. Fluid recurred once again and remained exudative in nature. Extensive work up revealed no causation for the effusion. There was no recurrence following the second thoracentesis for the follow-up of 1 year.

### Case 3

A 10-year-old asymptomatic boy was referred for a right-sided pleural effusion. When 40 days old, the boy had undergone surgery for a meningomyelocele and a VPS was placed for hydrocephalus. During the following one year, the shunt was replaced eight times due to its dysfunction. Eventually, at the age of one year, the location of the shunt was switched to the right atrium (VAS) and once again to ventriculo-vesical (VV) position at age 2. The shunt in the former location led to infective endocarditis and a right atrial thrombus formation requiring antimicrobial treatment and surgical extraction. At the age of 4, the VV shunt was once again replaced for malfunction. A year later, distal tip of the shunt was noticed at the external urethral meatus and the devise was removed. At this stage (age 5), VPLS was placed. During a routine renal ultrasound at the age 10, a moderate size right pleural effusion was observed. As the patient was asymptomatic, he was closely followed until the fluid resolved in a month; no thoracentesis was performed.

### Case 4

A 47-year-old male, a life-long non-smoker, was hospitalized with vertigo and the weakness of the lower extremities. He suffered with meningitis at age 6 and developed hydrocephalus. Patient had no prior pulmonary history. At the age of 46, for worsening hydrocephalus, he initially underwent VPS placement, but it failed hence an endoscopic third ventriculostomy was performed. However, a mechanical problem persisted at the proximal end of the catheter. Finally, the catheter was placed into the pleural space (VPLS). During the follow-up period of 4 years, no complications were observed.

### Case 5

A nine-year-old girl had undergone a resection of a meningomyelocele when she was 2 months old. Following the surgery a VPS was placed for hydrocephalus, which required three revisions for various reasons with no residual complications. At the age of four, she developed chronic renal failure due to retrograde uroflow, which required continuous peritoneal dialysis. During the course of peritoneal dialysis, she developed peritonitis and the catheter had to be removed and repositioned into the right atrium (VAS). The VAS led to a right atrial thrombus formation and it was replaced with VPLS. She developed no pulmonary complications for the latter device until her demise at the age of 9 years from unrelated reasons.

Characteristics of all the patients are depicted in Table 1.

**Table 1:** Characteristics of the patients

Pt	Age	Sex	Primary diagnosis	Onset of PE	Management	Symptom-free duration (Y)
1	74	F	Hypophysis macroadenoma	5 days	Thora X3	2
2	75	F	Lumber stabilization	20 days	Thora X2	1
3	10	M	Meningo-myelocele	5 years	Follow-up	5
4	47	M	Meningitis	-	Follow-up	5
5	9	F	Meningo-myelocele	-	No treatment	5

PE = Pleural effusion, Thora = Thoracentesis, Y = year

## Discussion

In patients with hydrocephalus, CSF can be shunted into any of the bodily cavities; however, peritoneal, atrial and the pleural locations are the most preferred ones. For the physician caring for such patients, it is imperative to be aware of shunt-related complications.

While CSF shunting procedures have significantly lowered the morbidity and mortality due to hydrocephalus, it has been estimated that 40–50% of children and up 29% of the adults will experience a failure of the shunt within the first year. Clinical indicators of early shunt failure include nausea, vomiting, irritability, altered consciousness, bulging of fontanels among infants; while depressed level of consciousness and loss of milestones are the main indicators of a late shunt failure.[10] Shunt-related complications can be divided into three categories: Mechanical failures, functional failures and infections.

Mechanical failures are the complications, which are related to either improper functioning of the shunt or improper placement of the device. Some of the common examples of this type of complication include obstruction of the shunt, fracture or disconnection of the device components and migration. The most common mechanical complication is obstruction of the shunt system, which presents with signs and symptoms of increased intracranial pressure (ICP). This may occur at any stage following the insertion and at any point along the shunt system.[11] The most common cause of obstruction is the in-growth of the portions of the choroid plexus or the ependymal surface of the ventricle into the inlet holes of the proximal catheter. Another reason could be the obstruction of the shunt valve with blood or cellular debris. Migration of the shunt can occur from the site of its initial placement into a position where it can no longer effectively drain the CSF.

Cerebrospinal fluid malabsorption may lead to abnormal accumulation of the fluid and may result in functional failure of the shunt. An abdominal pseudocyst may lead a peritoneal fluid collection around the peritoneal catheter and it may get infected and result in a functional failure.[11]

Infections are an important cause of shunt malfunction with a rate of 8–12% with most of these events occurring within 6 months of shunt insertion or shunt revision surgery. Most of these infections are thought to be due to inoculation with skin flora at the time of surgery or related to seeding from sites of distal infection.[11]

Because of its technical simplicity and high efficacy, plus the lower rate and lesser severity of its complications, VPS remains the procedure of choice for hydrocephalus.[12] A variety of complex abdominal conditions such as adhesions or history of peritonitis may render the peritoneal cavity a suboptimal location for CSF diversion. Ventriculo-peritoneal shunt is also prone to a variety of rare yet serious thoracic complications such as migration of the distal catheter to pulmonary artery,[13] broncho-pleural fistula, CSF hydrothorax or tension hydrothorax.[14] Nevertheless, at our institution VPS is preferred for most cases of hydrocephalus due to the large absorption surface of the peritoneal lining, ease of insertion, the low complication rate and accumulated experience. However, in cases of a prior major abdominal surgery, history of peritonitis, ascites, peritoneal dialysis and failure of prior VPS, we seek other alternatives for the shunt placement.

Ventriculo-atrial shunts have also been utilized in the past rather frequently; however, it is associated with significant morbidity and mortality.[8] Its most commonly reported complications include shunt nephritis (a rare, late and specific complication which is an immune-complex disease and occurs as a result of persistent infection of the VAS by an organism of low virulence),[15] pulmonary embolism, shunt infection and need for frequent revisions. Hence, its use has been declining over the past decade.[8] Multiple such serious complications were encountered in one of our patients who eventually required replacement of the shunt to an alternative site. We also observed that atrial thrombus and infective endocarditis are the main drawbacks of this device.

Ventriculo-pleural shunts have been used infrequently in the management of hydrocephalus. In the treatment of hydrocephalus at our institute, VPLS has become the ‘next preferred procedure’ in case if the VPS fails, due to its low complication rates, ease of insertion and interest and collaboration of the thoracic surgery team.

The use of VPLS was initially reported by Heile in 1914[16] as a temporary absorptive surface while managing shunts infections involving other sites and for the decompression of acute hydrocephalus from an intracranial tumor. In 1954, Ransohoff reported a series of 6 patients with tumor-induced hydrocephalus, who were successfully treated with a VPLS.[1] In a follow-up study, involving 85 children who were treated with VPLS, Ransohoff reported an overall success rate of 65%.[17] Even though initially rewarding, the long-term results were less satisfactory; shunt obstruction or pleural effusions are mostly developed in 3 years. Despite subsequent modification in the valve design, Venes *et al*. reported about 6 patients who developed large effusions following VPLS placement.[3]

In 1988, Jones reported a series of 29 children who were treated with VPLS in which only 7 shunts worked for more than a year. Three patients developed shunt infection, in four patients catheters became blocked by adhesions, one required substitution with VPS for a large recurrent symptomatic effusion while one patient in whom the shunt was functioning, died of unrelated causes.[18] On the contrary, Portnoy reported a series of 52 patients who were managed with VPLS with an anti-siphon device. In his study, only one of the 52 children required revision secondary to symptomatic pleural effusion.[19] Anti-siphon device is designed to help prevent the excessive drainage of CSF, which may be induced by the siphoning effect of hydrostatic pressure created by elevation of the ventricular catheter with respect to the distal catheter (i.e., when the patient sits, stands or is held erect).[20] Pressure-controlled device refers to a device designed to integrate a pressure range control valve, which drains the CSF from the ventricles to the peritoneum, atrium or pleura when the intraventricular pressure exceeds the desirable limits; likewise it blocks the drainage liquid allowing its pressure to be kept at the required physiological levels.[21] We did not use these anti-siphon devices, because the shunts we used were pressure-controlled devices.

In their review of 1500 patients, Hoffman *et al*.[22] analyzed 59 patients, who had received a VPLS. The most common indication for use of the pleural cavity was pre-existing VPS infection. In his series, 20% were found to have symptomatic pleural effusion that required revision, one-half of those were infants. The incorporation of an anti-siphon device in the shunt system seemed to decrease the possibility of a pleural effusion. Twenty-three of the 59 patients continue to function on their inserted VPLS, and in 9 of these the shunts have been functioning for over 5 years. They concluded that, VPLS seems to be a safe and simple form of diversionary CSF bypass. The risk of pleural effusion seemed to be highest among the infants, but was also observed at any age. Anti-siphon device seemed to reduce incidences of pleural effusion. We suspect that relatively small pleural surface in infants might be responsible for higher risk of pleural effusion.[23]

In a retrospective study, Megison *et al*.[24] reported use of 88 VPLS procedures on adults. Their overall complication rate was 24% (21 patients in 88). These included subdural effusions (SDEs) and hematoma in 5 patients (5.6%), proximal shunt obstruction in 6 patients (6.8%), pleural effusions in 4 (4.5%), dislodgement of distal catheter in 2 patients (2.2%), pleural adhesions prohibiting placement in 1 patient (1.2%), shunt infection in 2 patients (2.2%) and pneumothorax in one (1.1%). Subdural effusion refers to an effusion in the subdural space, usually of CSF.[25] Normal intraventricular pressure ranges from 30 to 155 mm H2O in sitting posture. On insertion of VPS, this drops to an average of 25 mm H_2_O in adults. This low pressure causes the brain to sag away from the calvarium, opening up the subdural space. Vessels traversing the subdural space are stretched resulting in transudation of fluid from the intravascular compartment to the subdural space. This is the probable pathogenesis of SDE in such cases.[26] They concluded that VPLS, when used with due precautions and careful patient selection, is a viable alternative for the treatment of adult hydrocephalus.

Symptomatic pleural collection of CSF is a rare complication in hydrocephalic children and adults treated with VPLS.[8] Only two of our five patients ever developed symptomatic effusion. The ability of pleural surfaces to absorb any accumulated fluid within the pleural cavity partially determines the occurrence and the degree of pleural fluid accumulation. Fluid accumulates if the rate of fluid formation exceeds the rate of absorption. Two hypotheses can explain the hydrothorax complicating the VPLS: (a) impaired pleural absorptive capacity, due to pleural damage secondary to prior infection and/or chronic exposure to CSF and (b) excessive drainage of CSF into the pleural space.[9] A symptomatic pleural effusion can occur at anytime during the course, as a result of a change in valve pressure or absorptive capacity of the parietal pleura, as seen in one of our patients, at 5 years.

A small asymptomatic pleural effusion is typically visible on the CXR, indicating that the VPLS is in action, but does not imply that it is dysfunctional. Thus, the CXR may be normal or show pleural effusions of varying sizes.

On the pleural fluid analyses, the fluid is usually a clear, transudate with a paucity of mononuclear cells, mimicking CSF. Only with infection there is a neutrophilic leukocytosis.[27] Pleural fluid eosinophilia has also been reported in three children with peripheral eosinophilia; in 2, however the mechanism of eosinophilia remained obscure.[28] Pleural fluid analysis is however non-specific. We are unable to explain lymphocytosis seen on the pleural fluid in one of our patients.

The diagnosis of VPLS obstruction in adults with hydrocephalus is often based on worsening clinical symptoms. The most common causes of shunt occlusion include accumulation of debris within the shunt catheter or adhesions or fibrous tissue blocking the distal catheter tip.[29] We suspect that in the immediate post-procedure period, accumulation of the debris within the shunt, especially the blood might have caused large effusions. This resolved spontaneously with gradual clearing of the debris and the patient no longer required repeated thoracentesis. Most adults with the shunt obstruction are ambulatory; however, pleural CSF collection may result in respiratory distress and thoracentesis may be required. In some patients with recurrent pleural effusions, definitive surgical treatment may be necessary.[8] Carrion *et al*. have demonstrated that administration of acetazolamide reduces CSF production, which may increase tolerance to VPLS and reduce respiratory symptoms.[30]

In conclusion, VPLS seems to be a safe and simple form of diversionary CSF bypass. However, symptomatic pleural effusion is a recognized complication. In most cases, the fluid resolves spontaneously seldom requiring specific treatment. The risk of pleural effusion seems to be highest in infants yet can occur at any age. Although the VPLS may not be the primary option in the management of hydrocephalus, it seems to be a reasonable alternative to VPS in older children and adults. Addition of anti-siphon device seems to prevent CSF accumulation in the pleural space.[31] Use of acetazolamide may reduce the CSF production and increase VPLS tolerance.
